# ADHESIVE CAPSULITIS EMBOLIZATION: A TECHNIQUE FOR THE FUTURE

**DOI:** 10.51894/001c.123115

**Published:** 2024-08-30

**Authors:** John P. Karns

**Affiliations:** 1 College of Osteopathic Medicne Michigan State University

## 92

### INTRODUCTION

Adhesive capsulitis embolization (ACE) is a promising treatment for refractory frozen shoulder, caused by inflammation, neovascularization, and fibrosis. ACE, targeting the neovascularized arterial supply, offers potential relief and improved function in patients unresponsive to conservative treatments. Recent clinical trials report significant improvements in shoulder function, pain, and daily activities at 1, 3, and 6 months, showcasing a 100% success rate without major adverse events. Therefore, ACE offers a potential alternative to more invasive techniques for the treatment of refractory adhesive capsulitis.

### OBJECTIVE

To describe pre-procedural and intra-procedural technical aspects of ACE to treat recurrent ACE.

### METHODS

A systematic search of the relevant scientific literature was performed. Items included in the search included “frozen shoulder”, “adhesive capsulitis”, and “embolization”, in addition to word variations. Relevant articles were screened and reviewed and relevant data were extracted. A technique guide was developed for image-guided ACE for the treatment of refractory adhesive capsulitis.

### RESULTS

The radial artery should undergo pre-procedural assessment and accessed with serial dilation utilizing the Seldinger technique. Access should be made using a 4-F sheath or smaller and upper extremity digital subtraction angiography should be performed from both axillary and subclavian arteries to identify all aberrant vasculature. Selective angiography should be performed and abnormal vasculature identified via lack of tubular structure. The embolic solution should be administered until blood flow stagnates on repeat angiography. Embolization end point should demonstrate the absence of hypervascularity with continued patency of the target vessel with anterograde flow. Technical challenges involving accessing the radial artery include occlusion, arterial spasm, and pseudoaneurysm. Complications are rare and often self-resolving but include skin discoloration, pruritus, and skin necrosis.

### DISCUSSION/CONCLUSIONS

In summary, adhesive capsulitis embolization (ACE) is a potential treatment for refractory frozen shoulder. Successful outcomes hinge on a thorough understanding of arterial anatomy, embolic endpoints, and technical challenges. The correct interpretation of angiographic findings and avoiding non-target embolization are crucial for ACE success. Despite minor complications, evidence supports ACE’s efficacy, hinting at its potential as a treatment for persistent adhesive capsulitis. ACE shows promise across diverse patient profiles, offering significant pain reduction within a year of assessment.

